# Contralateral fatigue during severe-intensity single-leg exercise: influence of acute acetaminophen ingestion

**DOI:** 10.1152/ajpregu.00084.2019

**Published:** 2019-05-29

**Authors:** Paul T. Morgan, Stephen J. Bailey, Rhys A. Banks, Jonathan Fulford, Anni Vanhatalo, Andrew M. Jones

**Affiliations:** ^1^Department of Sport and Health Sciences, College of Life and Environmental Sciences, University of Exeter, St. Luke’s Campus, Exeter, United Kingdom; ^2^Peninsula Clinical Research Facility, National Institute for Health Research, College of Medicine and Health, Exeter, United Kingdom

**Keywords:** intramuscular metabolites, intramuscular substrates, nonlocal muscle fatigue, ^31^P-magnetic resonance spectroscopy, Paracetamol

## Abstract

Exhaustive single-leg exercise has been suggested to reduce time to task failure (T_lim_) during subsequent exercise in the contralateral leg by exacerbating central fatigue development. We investigated the influence of acetaminophen (ACT), an analgesic that may blunt central fatigue development, on T_lim_ during single-leg exercise completed with and without prior fatiguing exercise of the contralateral leg. Fourteen recreationally active men performed single-leg severe-intensity knee-extensor exercise to T_lim_ on the left (Leg_1_) and right (Leg_2_) legs without prior contralateral fatigue and on Leg_2_ immediately following Leg_1_ (Leg_2-CONTRA_). The tests were completed following ingestion of 1-g ACT or maltodextrin [placebo (PL)] capsules. Intramuscular phosphorus-containing metabolites and substrates and muscle activation were assessed using ^31^P-MRS and electromyography, respectively. T_lim_ was not different between Leg_1ACT_ and Leg_1PL_ conditions (402 ± 101 vs. 390 ± 106 s, *P* = 0.11). There was also no difference in T_lim_ between Leg_2ACT-CONTRA_ and Leg_2PL-CONTRA_ (324 ± 85 vs. 311 ± 92 s, *P* = 0.10), but T_lim_ was shorter in Leg_2ACT-CONTRA_ and Leg_2PL-CONTRA_ than in Leg_2CON_ (385 ± 104 s, both *P* < 0.05). There were no differences in intramuscular phosphorus-containing metabolites and substrates or muscle activation between Leg_1ACT_ and Leg_1PL_ and between Leg_2ACT-CONTRA_ and Leg_2PL-CONTRA_ (all *P* > 0.05). These findings suggest that levels of metabolic perturbation and muscle activation at T_lim_ are not different during single-leg severe-intensity knee-extensor exercise completed with or without prior fatiguing exercise of the contralateral leg. Despite contralateral fatigue, ACT ingestion did not alter neuromuscular responses, muscle metabolites, or exercise performance.

## INTRODUCTION

The mechanisms of exercise-induced fatigue can be attributed to processes within the central nervous system, termed central fatigue, and within the contractile elements of the working muscle, termed peripheral fatigue. It is now recognized that peripheral and central fatigue development are interlinked, in part, via group III/IV muscle afferent feedback (25). Empirical support for a role of group III/IV muscle afferent feedback in modulating the mechanisms of neuromuscular fatigue is provided by reports that inhibition of group III/IV muscle afferent feedback, via lumbar intrathecal administration of fentanyl, lowers central fatigue development and results in increased skeletal muscle metabolic perturbation [greater and/or more rapid increases in ADP and Pi accumulation and declines in phosphocreatine (PCr) and pH] and, thus, peripheral fatigue development (1, 2, 8, 10–12, 39–41). Conversely, prior fatiguing single-limb exercise has been reported to accentuate central fatigue development and lead to lower peripheral fatigue development during subsequent fatiguing exercise in a contralateral or nonlocal (previously rested) muscle group, when group III/IV muscle afferent feedback would be expected to be elevated (3, 22, 23, 26, 34, 41). However, the underlying mechanisms of nonlocal muscle fatigue, including the effect of prior fatiguing single-limb exercise on skeletal muscle metabolic perturbation during subsequent fatiguing exercise in a contralateral or nonlocal muscle group, have yet to be resolved (see Ref. 23 for review). Moreover, while lumbar intrathecal administration of fentanyl and prior fatigue of a contralateral or nonlocal muscle group can alter group III/IV muscle afferent feedback and the physiological bases of exercise-induced neuromuscular fatigue (1–3, 8, 10–12, 22, 26, 28, 29, 34), the effect of such interventions on exercise performance is equivocal. Specifically, while some studies indicate that exercise performance is altered in these situations, i.e., enhanced with fentanyl (3) or impaired following contralateral fatigue (14, 22, 29, 34, 42), others report no significant effect (1, 2, 8, 10, 12, 16, 21, 35, 43, 46).

An emerging body of evidence suggests that oral ingestion of acetaminophen (ACT) can blunt the development of exercise-induced neuromuscular fatigue and improve exercise capacity and/or performance (19, 30–32). It is generally accepted that the principal mechanism of action of ACT is the inhibition of cyclooxygenase, the enzyme that catalyzes the synthesis of prostaglandins from arachidonic acid (4). Since prostaglandins sensitize nociceptors (37, 38) and since blocking cyclooxygenase attenuates group III/IV muscle afferent discharge during dynamic exercise (24), these mechanisms might account for reports of increased work output for the same level of perceived pain and exertion (19, 30) and elevated muscle activation (31, 32) during exercise after ACT ingestion. Therefore, ACT administration might be ergogenic by reducing, but not abolishing, the net magnitude of group III/IV muscle afferent feedback, leading to a blunting of exercise-induced central fatigue. Since ACT appears to attenuate exercise-induced neuromuscular fatigue by abating aspects of central fatigue development (19, 30–32), ACT might be more effective at lowering exercise-induced neuromuscular fatigue following prior exhaustive exercise in a contralateral limb. However, the effects of ACT ingestion on exercise-induced fatigue development and its underlying mechanisms following prior exercise in a contralateral limb have yet to be investigated.

The purpose of this study was to investigate the effects of ACT ingestion on exercise-induced neuromuscular fatigue and some of its underlying mechanisms during single-leg severe-intensity knee-extensor exercise completed with and without prior exhaustive severe-intensity knee-extensor exercise in the contralateral leg. It was hypothesized that *1*) prior exhaustive exercise would impair subsequent exercise tolerance in the contralateral leg by lowering muscle activation and the degree of muscle metabolic perturbation [changes in muscle pH and PCr ([PCr]), ADP ([ADP]), and Pi ([Pi]) concentrations] that could be attained, *2*) ACT ingestion would enhance single-leg knee-extensor exercise tolerance by increasing muscle activation [higher surface electromyogram (EMG)] and permitting a greater degree of muscle metabolic perturbation, and *3*) completion of prior exercise by the contralateral leg would lead to a greater enhancement of exercise tolerance following ACT ingestion.

## MATERIALS AND METHODS

### 

#### Subjects.

Fourteen active men [age 23.8 (SD 4.7) yr, height 1.80 (SD 0.10) m, body mass 81.6 (SD 14.9) kg] volunteered to participate in the study. All procedures were approved by the Ethics Committee of the Department of Sport and Health Sciences, University of Exeter. The study conformed to the principles of the World Medical Association Declaration of Helsinki. Subjects completed a health questionnaire that was checked by a medical doctor to ensure that the subjects could safely consume ACT before performing exhaustive exercise. The questionnaire incorporated questions pertaining to known allergies to medications, current intake of medication, and prior use of ACT, as well as any history of illnesses, cigarette and illegal drug use, alcohol consumption, and chronic illnesses (personal and family history). Before each visit, subjects were required to refrain from caffeine (for ≥12 h), strenuous exercise and alcohol (for ≥24 h), and analgesics and any form of anti-inflammatory drug (for the duration of the experiment) and to arrive in a fully rested, hydrated state. With the exception of these restrictions, subjects were instructed to maintain their usual diet and exercise regimen during the study. All tests were performed at a similar time of day (±2 h).

#### Preexperimental procedures.

**Fig. 1.**
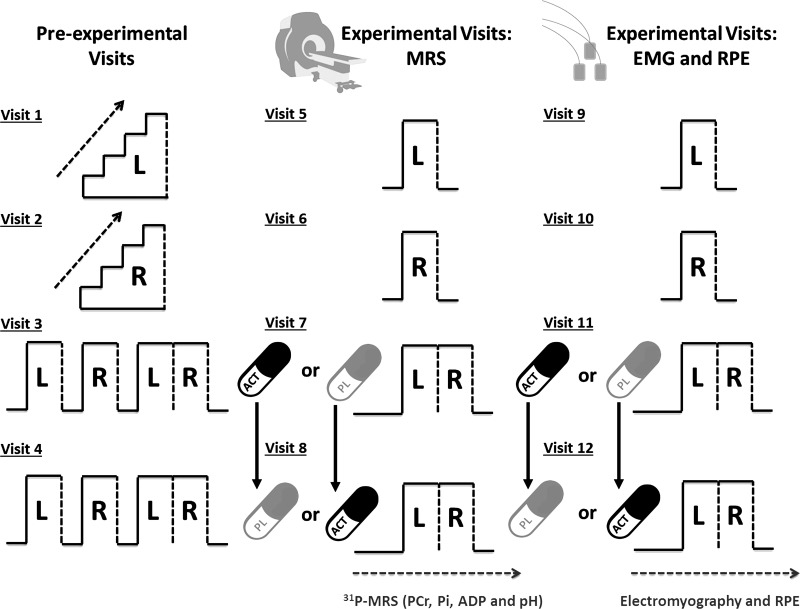
Protocol schematic. *Visits 1–4* were completed within a replica of the MRI scanner. Subjects completed a single-leg incremental test on the left (L) leg (*visit 1*, Leg_1_) and right (R) leg (*visit 2*, Leg_2_). On *visits 3* and *4*, subjects completed a familiarization session, which comprised a single-leg severe-intensity constant work rate (CWR) test to task failure with Leg_1_ and Leg_2_ and a crossover test where the Leg_1_ protocol was repeated and immediately followed by the Leg_2_ protocol (interspersed with 10 min of passive recovery). During *visits 5* and *6*, subjects completed the Leg_1_ and Leg_2_ protocols, respectively, without oral consumption of capsules. On *visits 7* and *8*, subjects commenced the crossover test 45 min following consumption of 1 g of maltodextrin (PL) and 45 min following consumption of 1 g of acetaminophen (ACT). *Visits 5–8* were completed within the bore of an MRI scanner for assessment of intramuscular phosphorus-containing substrates and metabolites and then replicated within a replica of the MRI scanner (*visits 9–12*) to assess muscle electromyography (EMG) and ratings of perceived exertion (RPE). Dashed vertical lines represent the limit of tolerance [i.e., time to task failure (T_lim_)] for each trial and/or leg. MRS, magnetic resonance spectroscopy; PCr, phosphocreatine.

Subjects visited the laboratory on 12 occasions over an 8- to 12-wk period to complete the experimental testing, with ≥72 h separating consecutive tests (Fig. 1). The experimental testing incorporated four preexperimental trials (*visits 1–4*) and eight experimental trials (*visits 5–12*). *Visits 1–4* were completed within a replica of an MRI scanner (with no magnetic field present). Initially, subjects completed a single-limb incremental test on the left leg (*visit 1,* Leg_1_) and right leg (*visit 2,* Leg_2_) to task failure to establish the limb-specific work rates that would be applied in subsequent experimental visits (see below). After these preliminary tests, subjects completed a familiarization session on *visits 3* and *4* that comprised a single-leg severe-intensity constant work rate (CWR) test to task failure with the left leg (Leg_1_), a single-leg severe-intensity CWR test to task failure with the right leg (Leg_2_), and a crossover test, where the Leg_1_ protocol was repeated and immediately followed by the trial to assess contralateral fatigue in Leg_2_ (Leg_2-CONTRA_ protocol). Severe-intensity exercise is defined as being above critical power (33). In these preliminary tests, the Leg_1_, Leg_2_, and Leg_2_ contralateral (Leg_2-CONTRA_) protocols were interspersed with 10 min of passive recovery.

#### Experimental procedures.

During *visits 5* and *6*, subjects completed the Leg_1_ and Leg_2_ protocols without oral consumption of capsules (Leg_1CON_ and Leg_2CON_, respectively). On *visits 7* and *8*, subjects completed the crossover limb tests described above 45 min following consumption of 1 g of maltodextrin [placebo (PL)] to determine time to task failure (T_lim_) values for Leg_1_ (Leg_1PL_) and Leg_2-CONTRA_ (Leg_2PL-CONTRA_) and 45 min following consumption of 1 g of ACT to determine T_lim_ values for Leg_1_ (Leg_1ACT_) and Leg_2-CONTRA_ (Leg_2ACT-CONTRA_). PL and ACT were administered in the form of two identically colored capsules. PL consisted of maltodextrin powder in gelatin capsules designed to have an appearance similar to ACT without analgesic or antipyretic effects. The oral consumption of PL and ACT ~45 min before commencement of exercise was selected to broadly coincide with attainment of the peak plasma ACT concentration ([ACT]), which occurs ~60 min after ACT ingestion (4, 17), at the onset of the Leg_2-CONTRA_ tests. The PL and ACT conditions were administered double-blind in a counterbalanced crossover experimental design. *Visits 5–8* were completed within the bore of an MRI scanner for assessment of exercise-induced changes in intramuscular phosphorus-containing substrates and metabolites. *Visits 5–8* were replicated in *visits 9–12* within a replica of the MRI scanner (with no magnetic field present) to assess muscle EMG and ratings of perceived exertion (RPE).

#### Experimental setup.

Exercise tests were performed with subjects in a prone position within the bore of a 1.5-T superconducting magnet (Gyroscan Clinical Intera, Philips, The Netherlands) using a custom-built ergometer for assessment of intramuscular [PCr], [Pi], [ADP], and pH (*visits 5–8*) or within a replica of the MRI scanner for preliminary testing (*visits 1–4*) and assessment of EMG and RPE responses (*visits 9–12*). The subject’s feet were fastened securely to padded foot braces using Velcro straps and connected to the ergometer load baskets via a rope-and-pulley system. The sprocket arrangement was such that when a bucket containing nonmagnetic weights was attached, it provided a concentric-only resistive load, allowing for the performance of rhythmic knee-extension exercise. Single-leg knee extensions over a distance of ∼0.22 m were performed continuously at a constant frequency, which was set in unison with the magnetic pulse sequence (40 pulses/min) to ensure that the quadriceps muscle was in the same phase of contraction during each magnetic resonance pulse acquisition. To prevent displacement of the quadriceps relative to the magnetic resonance spectroscopy (MRS) coil, Velcro straps were also fastened over the subject's thighs, hips, and lower back.

#### Experimental protocol.

To determine peak work rate for each leg, the subjects initially completed single-leg incremental knee-extensor exercise on *visits 1* and *2* until they were unable to continue the prescribed work rate, as described previously (44). The load for the initial increment was 4 kg, which was increased by 0.5 kg/min thereafter until T_lim_. T_lim_ was recorded when the subjects were unable to sustain the required contraction frequency for three consecutive repetitions. After these initial tests, the subjects were familiarized with the different exercise tests that comprised the experimental testing protocol. During these visits, a limb-specific, severe-intensity work rate, which was expected to elicit T_lim_ in ~5–8 min, was prescribed for each subject. The work rate initially selected was 80% of the peak work rate attained in the incremental test, and depending on responses in the familiarization tests, this was adjusted for each individual to give the desired exercise duration during subsequent tests.

The experimental exercise protocol consisted of CWR single-leg severe-intensity knee extension to T_lim_. Initially, the subjects completed single-leg knee-extension exercise for each limb individually over two separate laboratory visits. Subsequently, to investigate the influence of ACT on contralateral leg fatigue, the subjects completed single-leg knee-extension exercise until task failure with Leg_1_ followed consecutively (<3 s) by the identical task with the contralateral leg (i.e., Leg_2_). These crossover tests to assess contralateral fatigue in Leg_2_ were completed 60 min following consumption of PL and ACT over two separate laboratory visits. For all trials, the subjects received strong verbal encouragement to continue for as long as possible, but they were given no feedback on the elapsed time.

#### MRS measurements.

^31^P-MRS data, with a spectral width of 1,500 Hz and 1,000 data points, were acquired every 1.5 s. Phase cycling with four phase cycles led to a spectrum being acquired every 6 s. The subsequent spectra were quantified by peak fitting using the AMARES fitting algorithm in the jMRUI (v3) software package. Absolute values of [PCr] and [Pi] were subsequently calculated from the PCr-to-ATP and Pi-to-ATP ratios, with the assumption of 8.2 mM ATP. Intracellular pH was calculated using the chemical shift of the Pi spectra relative to the PCr peak. [ADP] was calculated as described by Kemp et al. (27). In all cases, relative amplitudes were corrected for partial saturation resulting from the short repetition time relative to T1 relaxation time via a spectrum consisting of 24 averages that was acquired with a TR of 20 s before the commencement of exercise testing.

#### Electromyography.

Throughout *visits 9–12*, muscle activity of the right and left vastus lateralis was recorded using active bipolar bar electrodes with a single differential configuration (model DE2.1, DelSys, Boston, MA). Initially, the leg was shaved and cleaned with alcohol to minimize skin impedance. The electrodes were placed over the respective muscle bellies parallel to the longitudinal axis of each muscle (Surface EMG for Non-Invasive Assessment of Muscles guidelines). Double-sided adhesive tape and a hypoallergenic medical tape were used to ensure stability of the EMG sensor. The position of the EMG electrodes was measured with respect to the location of the patella and the anterior superior iliac spine and marked with indelible ink to ensure placement in the same location on subsequent visits. The ground electrode was placed over the patella of the respective leg. The EMG signals were preamplified (×1,000), band-pass-filtered (20–450 Hz; Bagnoli-8, DelSys), and then transferred to a computer with a sampling frequency of 2 kHz. EMG data were recorded continuously and digitized synchronously with 16-bit resolution via an analog-to-digital converter (±5-V range, CED 1401 power, Cambridge Electronic Design, Cambridge, UK) using Spike2 software (Cambridge Electronic Design). During these trials, RPE was measured at 2-min intervals from the onset of exercise using Borg’s 6–20 scale (9).

#### Data analysis.

Baseline values for [PCr], [Pi], [ADP], and pH were defined as the mean values measured over the final 60 s of rest (i.e., before initiation of the severe-intensity exercise bout). Baseline values for Leg_2_ during the crossover protocol (for both PL and ACT) were calculated during the final 60 s of exhaustive Leg_1_ exercise. End-exercise values for these variables were defined as the mean values measured over the final 30 s of exercise. The changes (Δ) in [PCr], [Pi], [ADP], and pH across the protocol were then calculated as the difference between end-exercise and baseline values. [PCr], [Pi], and [ADP] are expressed as absolute concentrations and as percent change relative to resting baseline (i.e., 100%). The overall rate of change for [PCr], [Pi], [ADP], and pH was calculated as the difference between end-exercise and baseline values divided by T_lim_. EMG was average-rectified and normalized to the first 30 s of each trial (aEMG). For analysis, T_lim_ values obtained from *visits 5–8* were used. *Visits 9–12* were used to overlay EMG and RPE responses on ^31^P-MRS data.

#### Statistics.

Differences in T_lim_, baseline and end-exercise aEMG, and muscle [PCr], [Pi], [ADP], and pH between control limbs (i.e., Leg_1_ vs. Leg_2_) were assessed using paired-samples *t*-tests. A two-way (time × condition) repeated-measures ANOVA was employed to test for differences in the profiles of muscle [PCr], [Pi], [ADP], and pH, aEMG (using 30-s mean values), and RPE (using 120-s mean values). Where the ANOVA revealed a significant main or interaction effect, post hoc tests were completed using Bonferroni’s correction. For calculation of effect size, partial η^2^ was used for omnibus tests. Cohen's *d* was used to calculate the effect size for paired *t*-tests and post hoc comparisons. Where sphericity was violated, a Greenhouse-Geisser correction factor was applied. For all tests, results were considered statistically significant when *P* < 0.05. Data are presented as means (SD) unless otherwise indicated. All statistical analyses were conducted using IBM SPSS Statistics version 24.

## RESULTS

**Fig. 2.**
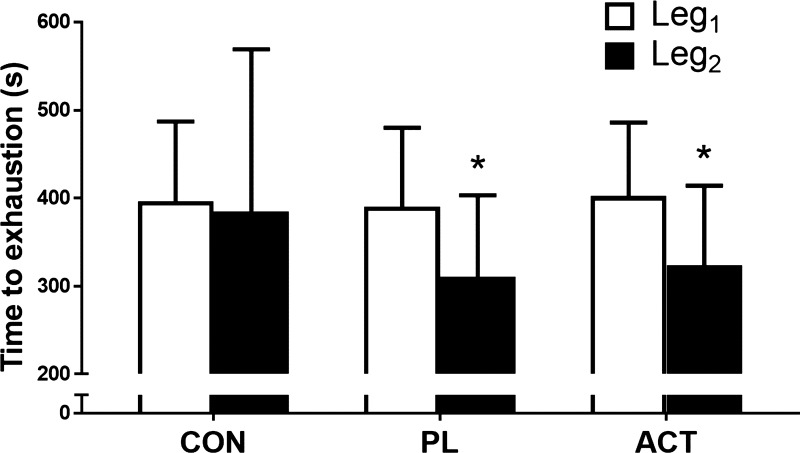
Exercise tolerance [time to task failure (exhaustion)] in Leg_1CON_, Leg_2CON_, Leg_1PL_, Leg_2PL-CONTRA_, Leg_1ACT_, and Leg_2ACT-CONTRA_ conditions. CON, control; PL, placebo; CONTRA, contralateral; ACT, acetaminophen. Values are means (SD). *Significantly different from Leg_1_ (*P* < 0.05).

**Fig. 3.**
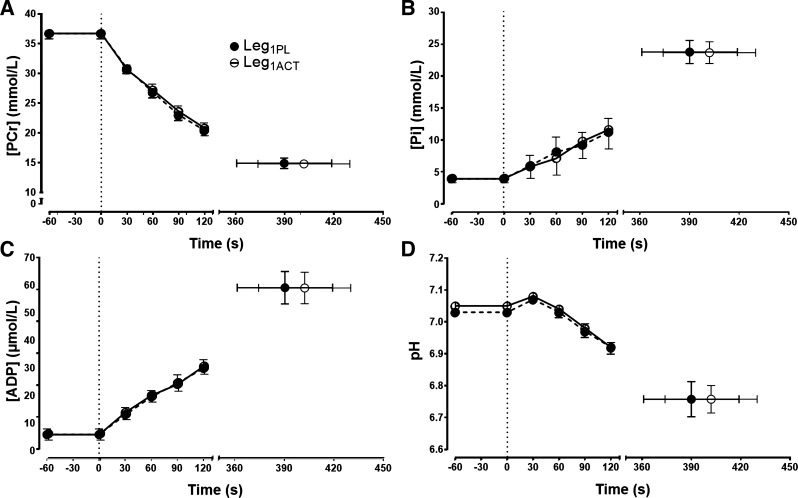
Intramuscular phosphocreatine (PCr) concentration ([PCr]; *A*), Pi concentration ([Pi]; *B*), ADP concentration ([ADP]; *C*), and pH (*D*) during severe-intensity single-leg knee-extensor exercise in the left leg following ingestion of placebo (Leg_1PL_) and acetaminophen (Leg_1ACT_). Values are group means ± SE.

There was no difference in T_lim_ during the Leg_1CON_ [396 (SD 105) s] and Leg_2CON_ [385 (SD 104) s] protocols [*P* = 0.20, *d* = 0.10, coefficient of variation = 2.0 (SD 1.7) %; Fig. 2]. Moreover, there were no differences in [PCr], [Pi], [ADP], pH (Table 1, Fig. 3), aEMG amplitude (Table 2, Fig. 5), and RPE (Fig. 6) between Leg_1CON_ and Leg_2CON_ at any time (all *P* > 0.05). Compared with Leg_2CON_, T_lim_ was reduced by 19% when Leg_2_ was preceded by exhaustive exercise in Leg_1_ following consumption of PL [Leg_2CON_ and Leg_2PL-CONTRA_ 385 (SD 104) and 311 (SD 92) s, respectively, *P* < 0.01, *d* = 0.76; Fig. 2].

**Table 1.** Muscle metabolic responses in Leg_1CON_, Leg_1PL_, Leg_1ACT_, Leg_2CON_, Leg_2PL-CONTRA_, and Leg_2ACT-CONTRA_ conditions

	Leg_1CON_	Leg_1PL_	Leg_1ACT_	Leg_2CON_	Leg_2PL-CONTRA_	Leg_2ACT-CONTRA_
[PCr]						
Baseline, %	100 (0)	100 (0)	100 (0)	100 (0)	92 (5)*	93 (4)*
120 s, %	70 (8)	70 (8)	71 (7)	71 (8)	62 (9)*	63 (7)*
End exercise, %	42 (9)	41 (9)	41 (8)	44 (8)	45 (7)	44 (8)
Rate of change, mmol/s	−0.06 (0.01)	−0.06 (0.03)	−0.06 (0.02)	−0.05 (0.03)	−0.06 (0.04)	−0.06 (0.03)
[Pi]						
Baseline, %	100 (0)	100 (0)	100 (0)	100 (0)	125 (24)*	126 (23)
120 s, %	310 (66)	313 (71)	306 (62)	312 (66)	316 (70)	318 (64)
End exercise, %	590 (149)	590 (137)	594 (156)	588 (177)	459 (110)*	460 (109)*
Rate of change, mmol/s	0.05 (0.02)	0.05 (0.02)	0.05 (0.02)	0.05 (0.02)	0.05 (0.02)	0.05 (0.02)
[ADP]						
Baseline, %	100 (0)	100 (0)	100 (0)	100 (0)	200 (78)*	201 (77)*
120 s, %	404 (161)	415 (183)	400 (148)	412 (170)	538 (176)	516 (154)*
End-exercise, %	1,028 (386)	1,036 (421)	1,046 (409)	1,024 (401)	980 (316)	978 (312)
Rate of change, µmol/s	0.15 (0.08)	0.15 (0.09)	0.14 (0.07)	0.15 (0.09)	0.17 (0.10)	0.15 (0.09)
pH						
Baseline	7.04 (0.01)	7.03 (0.02)	7.05 (0.04)	7.04 (0.03)	7.04 (0.03)	7.05 (0.02)
120 s	6.96 (0.09)	6.94 (0.07)	6.92 (0.08)	6.95 (0.08)	6.93 (0.10)	6.94 (0.08)
End-exercise	6.77 (0.18)	6.76 (0.15)	6.76 (0.16)	6.83 (0.15)	6.83 (0.20)	6.80 (0.15)

Values are means (SD) of 14 male subjects who performed single-leg severe-intensity knee-extensor exercise to task failure on the left (Leg_1_) and right (Leg_2_) legs without prior contralateral fatigue and on Leg_2_ immediately following Leg_1_ (Leg_2-CONTRA_). PL, placebo; ACT, acetaminophen; [PCr], phosphocreatine concentration; [Pi], Pi concentration; [ADP], ADP concentration.

* *P* < 0.05 vs. Leg_2CON_.

**Table 2.** EMG responses of the vastus lateralis in Leg_1CON_, Leg_1PL_, Leg_1ACT_, Leg_2CON_, Leg_2PL-CONTRA_, and Leg_2ACT-CONTRA_ conditions

	Leg_1CON_	Leg_1PL_	Leg_1ACT_	Leg_2CON_	Leg_2PL-CONTRA_	Leg_2ACT-CONTRA_
EMG_RMS_ amplitude						
Baseline, mV	0.04 (0.01)	0.04 (0.02)	0.04 (0.02)	0.04 (0.02)	0.05 (0.02)*	0.05 (0.02)*
End exercise, %	229 (54)	224 (43)	238 (51)	234 (52)	226 (58)	242 (52)
120 s, %	150 (27)	160 (25)	166 (26)	158 (29)	155 (32)	158 (34)

Values are means (SD) of 10 subjects who performed single-leg severe-intensity knee-extensor exercise to task failure on the left (Leg_1_) and right (Leg_2_) legs without prior contralateral fatigue and on Leg_2_ immediately following Leg_1_ (Leg_2-CONTRA_). PL, placebo; ACT, acetaminophen; RMS, root mean square.

* *P* < 0.05 vs. Leg_2CON_.

### 

#### Effect of ACT on single-leg exercise tolerance and contralateral leg fatigue.

There was no difference in T_lim_ between the Leg_1CON_ [396 (SD 105) s], Leg_1ACT_ [402 (SD 101) s], and Leg_1PL_ [390 (SD 106) s] conditions (*P* = 0.55, η^2^ = 0.07; Fig. 2). T_lim_ values were significantly lower for Leg_2PL-CONTRA_ and Leg_2ACT-CONTRA_ than for Leg_2CON_ (*P* < 0.01, η^2^ = 0.71; Fig. 2). However, there was no difference in T_lim_ between Leg_2PL-CONTRA_ and Leg_2ACT-CONTRA_ [311 (SD 92) and 324 (SD 85) s, respectively, *P* = 0.09, *d* = 0.15, coefficient of variation = 4.9 (SD 5.4) %; Fig. 2].

#### Muscle metabolic measurements.

**Fig. 4.**
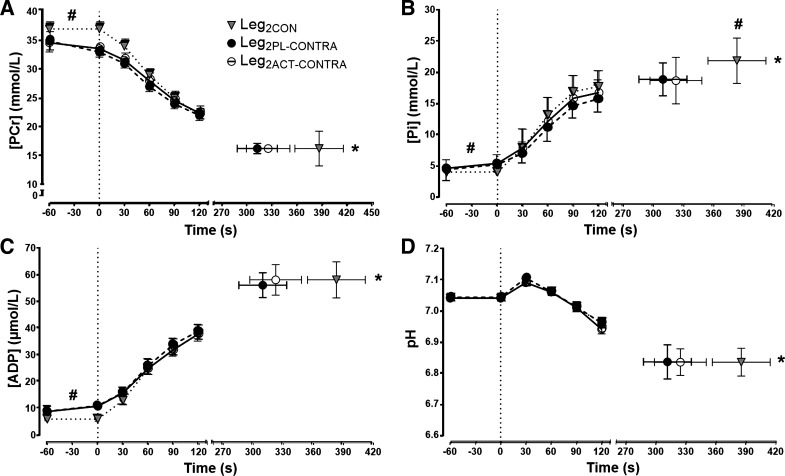
Intramuscular phosphocreatine (PCr) concentration ([PCr]; *A*), Pi concentration ([Pi]; *B*), ADP concentration ([ADP]; *C*), and pH (*D*) during severe-intensity single-leg knee-extensor exercise in the right control leg (Leg_2CON_) and in the right leg following prior exhaustive exercise in the left leg after ingestion of placebo (Leg_2PL-CONTRA_) and ACT (Leg_2ACT-CONTRA_). Values are group means ± SE. *Time to task failure (T_lim_) significantly different from Leg_2PL-CONTRA_ and Leg_2ACT-CONTRA_ (*P* < 0.05); #[Pi] significantly different from Leg_2PL-CONTRA_ and Leg_2ACT-CONTRA_ (*P* < 0.05).

The [PCr], [Pi], [ADP], and pH profiles are illustrated in Fig. 3 for Leg_1PL_ and Leg_1ACT_ and in Fig. 4 for Leg_2CON_, Leg_2PL-CONTRA_, and Leg_2ACT-CONTRA_. There were no significant differences in [PCr], [Pi], [ADP], or pH at any time points between Leg_1CON_ and Leg_2CON_ (all *P* > 0.05; Table 1, Fig. 3). Similarly, there were no significant differences in end-exercise [PCr], [ADP], and pH between the Leg_2CON_, Leg_2PL-CONTRA_, and Leg_2ACT-CONTRA_ conditions (Table 1, Fig. 4). However, end-exercise [Pi] was significantly lower in Leg_2PL-CONTRA_ and Leg_2ACT-CONTRA_ than in Leg_2CON_ (*P* < 0.05, η^2^ = 0.89; Table 1, Fig. 4). Baseline [PCr] was significantly higher (*P* < 0.0001, η^2^ = 3.04), and [Pi] (*P* < 0.01, η^2^ = 2.13) and [ADP] (*P* < 0.01, η^2^ = 2.55; Table 1, Fig. 4) were significantly lower, in Leg_2CON_ than Leg_2PL-CONTRA_ and Leg_2ACT-CONTRA_, respectively. The rates of change for [Pi], [PCr], [ADP], and pH were not different between Leg_2CON_, Leg_2PL-CONTRA_, and Leg_2ACT-CONTRA_ conditions.

#### Electromyography.

**Fig. 5.**
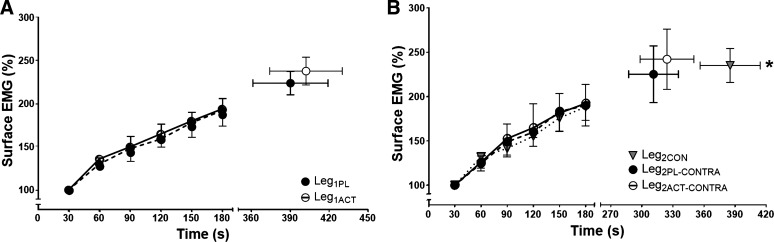
*A*: surface EMG of the vastus lateralis during severe-intensity single-leg knee-extensor exercise in the left leg following ingestion of placebo (Leg_1PL_) and acetaminophen (Leg_1ACT_). *B*: surface EMG of the vastus lateralis during severe-intensity single-leg knee-extensor exercise in the right control leg (Leg_2CON_) and in Leg_2_ following prior exhaustive exercise in Leg_1_ after ingestion of placebo (Leg_2PL-CONTRA_) and acetaminophen (Leg_2ACT-CONTRA_). Mean values for average rectified EMG during each muscle contraction were calculated and averaged over each 30-s period. Values are group means ± SE relative to the first 30 s of each trial. *Time to task failure (T_lim_) significantly different from Leg_2PL-CONTRA_ and Leg_2ACT-CONTRA_ (*P* < 0.05).

aEMG amplitude of vastus lateralis rose significantly from the first minute of exercise to end exercise in all conditions (*P* < 0.01, η^2^ = 3.8; Fig. 5). However, there were no differences in aEMG between Leg_1CON_, Leg_1PL_, and Leg_1ACT_ at T_lim_ (Table 2, Fig. 5). End-exercise aEMG in Leg_2CON_ was also not different from Leg_2PL-CONTRA_ and Leg_2ACT-CONTRA_ (Table 2, Fig. 5). However, absolute aEMG was elevated at the start of Leg_2PL-CONTRA_ and Leg_2ACT-CONTRA_ compared with Leg_2CON_ (*P* < 0.01, η^2^ = 0.58; Table 2, Fig. 5).

#### Ratings of perceived exertion.

**Fig. 6.**
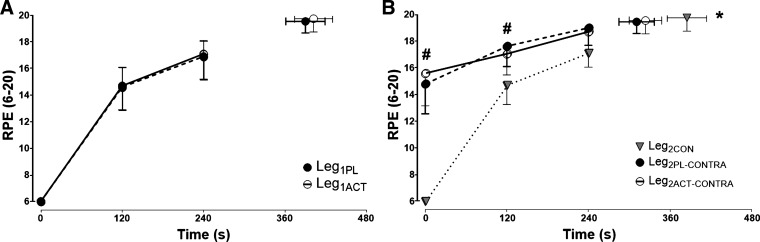
*A*: ratings of perceived exertion (RPE) during severe-intensity single-leg knee-extensor exercise of the left leg following ingestion of placebo (Leg_1PL_) and acetaminophen (Leg_1ACT_). *B*: RPE during severe-intensity single-leg knee-extensor exercise of the right control leg (Leg_2CON_) and the right leg following prior exhaustive exercise in the left leg after ingestion of placebo (Leg_2PL-CONTRA_) and acetaminophen (Leg_2ACT-CONTRA_). Values are group means ± SE. *Time to task failure (T_lim_) significantly different from Leg_2PL-CONTRA_ and Leg_2ACT-CONTRA_ (*P* < 0.05); #RPE significantly different from Leg_2CON_ (*P* < 0.05).

RPE increased in all trials following the onset of exercise (Fig. 6). However, there were no differences in RPE between Leg_1CON_, Leg_1PL_, and Leg_1ACT_ at any time point (*P* = 0.72, η^2^ = 0.08; Fig. 6). The rate of rise and the end-exercise RPE were also not different between the Leg_2CON_ trial and the Leg_2PL-CONTRA_ and Leg_2ACT-CONTRA_ trials (*P* = 0.66, η^2^ = 0.18). However, at the onset of exercise, RPE was significantly higher in Leg_2PL-CONTRA_ and Leg_2ACT-CONTRA_ than in Leg_2CON_ (*P* < 0.01, η^2^ = 0.55; Fig. 6). Specifically, during the first 2 min of exercise, RPE was elevated 14% and 13% in Leg_2PL-CONTRA_ and Leg_2ACT-CONTRA_, respectively, compared with Leg_2CON_ (*P* < 0.01). There were no differences in RPE at any time points between Leg_2PL-CONTRA_ and Leg_2ACT-CONTRA_ (*P* = 0.60, η^2^ = 0.21; Fig. 6).

## DISCUSSION

The principal original finding of this study was that while T_lim_ was lower during severe-intensity single-leg knee-extensor exercise after completion of prior fatiguing exercise in the contralateral leg, this effect was not mitigated by acute ACT ingestion. We found no differences in the rates of change or end-exercise values for skeletal muscle activation (via EMG), metabolic perturbation (via ^31^P-MRS), and perception of effort (via RPE) during exercise after prior contralateral leg fatigue following ACT and PL ingestion. Moreover, there were no differences in T_lim_, skeletal muscle activation, metabolic perturbation, and RPE during single-leg exercise without completion of prior fatiguing exercise by the contralateral leg following ACT and PL ingestion. These findings do not support our experimental hypotheses and suggest that acute ingestion of 1 g of ACT does not improve T_lim_, skeletal muscle activation, metabolic perturbation, or perceived exertion during single-leg severe-intensity knee-extensor exercise completed with or without prior fatiguing exercise by the contralateral leg.

In the present study, T_lim_ was shorter in the Leg_2PL-CONTRA_ than the Leg_2CON_ protocol, indicative of an earlier task failure after completion of exhaustive exercise in the contralateral leg compared with no prior fatiguing contralateral leg exercise. This observation is consistent with some (3, 14, 22, 29, 34, 42), but not all (16, 21, 35, 43, 46), previous studies reporting greater fatigue development after prior contralateral or nonlocal muscle fatigue. While the neuromuscular bases of contralateral fatigue development have yet to be fully resolved (23), there is evidence that greater central fatigue makes an important contribution to this phenomenon (3). In the current study, RPE was higher at baseline and over the initial stages of the Leg_2PL-CONTRA_ test than the Leg_2CON_ test, leading to an earlier attainment of peak RPE and T_lim_, consistent with previous observations (3) and the notion that afferent feedback may contribute to increased pain and effort sensation (1, 20). Amann et al. (3) reported a lower EMG response at task failure and reduced peripheral fatigue development after prior contralateral leg fatigue. Although the EMG amplitude was not different at task failure in the current study between the Leg_2CON_ and Leg_2PL-CONTRA_ tests, baseline EMG was elevated in the Leg_2PL-CONTRA_ condition, presumably due to isometric stabilization, leading to the earlier attainment of the same peak EMG amplitude. It should be noted here that EMG responses were normalized to the initial exercise values in the present study and in the study of Amann et al. (3). The greater muscle activation in the nonexercising contralateral leg during the baseline “resting” period in the Leg_2PL-CONTRA_ condition was accompanied by lower muscle [PCr] and higher muscle [Pi] and [ADP] than in the Leg_2CON_ condition. Since there were no differences in muscle [PCr] and [ADP] at T_lim_ and since the rates of change in [PCr] and [ADP] were not different between the Leg_2CON_ and Leg_2PL-CONTRA_ tests, the muscle [PCr] nadir and [ADP] peak were attained earlier in the Leg_2PL-CONTRA_ test. These observations cohere with reports that the end-exercise values of muscle [PCr], [ADP], and pH are consistent when several bouts of exhaustive exercise of differing duration are completed within the severe-intensity domain (7, 45) and when T_lim_ is altered via prior passive heating of the legs (6) or by hyperoxic gas inhalation (45). Interestingly, however, and despite a higher baseline muscle [Pi] in the Leg_2PL-CONTRA_ than the Leg_2CON_ condition, muscle [Pi] was lower at task failure in the Leg_2PL-CONTRA_ test. These novel observations suggest that the ergolytic effect of prior contralateral fatigue may be related, at least in part, to a limitation in the attainment of peak intramuscular [Pi].

It is unclear why prior contralateral leg fatigue limited the attainment of peak [Pi] in the Leg_2PL-CONTRA_ condition compared with the Leg_2CON_ condition, whereas the peak [ADP] and the nadir in pH and [PCr] were not different between these conditions. However, our observations of a limited peak perturbation of muscle [Pi], but not pH, [PCr], and [ADP], when group III/IV muscle afferent feedback would be expected to be elevated via prior contralateral fatigue (3) are in accord with studies from others who observed greater peak perturbation of muscle [Pi], but not pH, [PCr], and [ADP], when group III/IV muscle afferent feedback was abolished via lumbar intrathecal administration of fentanyl (8, 11, 12). Together, these complementary observations suggest that intramuscular phosphorus-containing metabolites and substrates may not respond in a uniform manner to manipulations in skeletal muscle group III/IV afferent feedback and that muscle [Pi] might be the more sensitive marker of muscle metabolic strain. However, it should be acknowledged that since within-test variability is greater for contracting skeletal muscle [Pi] than for pH, [PCr], and [ADP] (15), further research is required to verify these observations.

Although the completion of prior single-leg fatiguing exercise lowered T_lim_ during subsequent exercise in the contralateral leg in the current study, there were no differences between the Leg_2ACT-CONTRA_ and Leg_2PL-CONTRA_ conditions in T_lim_, RPE, or muscle activation and phosphorus-containing metabolites and substrates. Similarly, and also in contrast to our hypothesis, acute ACT ingestion did not alter T_lim_, RPE, or muscle activation, pH, [PCr], [ADP], or [Pi] during single-leg severe-intensity knee-extensor exercise completed without prior fatiguing exercise in the contralateral leg: these responses were similar between the Leg_1CON_, Leg_1PL_, and Leg_1ACT_ conditions. These findings conflict with reports that acute ACT consumption can improve exercise performance by increasing work output for the same level of pain and effort sensation (19, 30) and by increasing muscle activation (31, 32).

### 

#### Experimental considerations.

The lack of an ergogenic effect of ACT administration in the current study might be due to differences in the ACT administration procedure compared with previous studies reporting improved performance and delayed neuromuscular fatigue development (19, 30–32). In the present study, ACT was ingested 45 min before the start of the Leg_1ACT_ test, which immediately transitioned to the Leg_2ACT-CONTRA_ protocol, the primary focus of the current study. Since peak plasma [ACT] is attained ~60 min after oral ACT ingestion (4, 17), we elected to administer ACT such that peak plasma [ACT] was expected to coincide with the onset of the Leg_2ACT-CONTRA_, rather than the Leg_1ACT_, protocol. This might account for the lack of an ergogenic effect of ACT during the Leg_1ACT_ protocol compared with other studies in which ACT was administered 60 min before the performance trial (19, 30–32). Therefore, we cannot exclude the possibility that earlier ACT ingestion (18), at the same or a greater dose (19, 30), might have resulted in improved single-leg severe-intensity exercise tolerance. However, interstudy differences in participant characteristics (i.e., training status, motivation, and responsiveness to analgesic medication) may have contributed to the differences in ergogenicity observed following ACT ingestion between the current study and some previous studies (19, 30–32).

In addition to differences in the ACT dosing procedure, the lack of an ergogenic effect of ACT administration in the current study might be linked to the nature of the fatiguing exercise test administered. Our subjects completed continuous single-leg severe-intensity knee-extensor exercise until task failure with no predetermined end point (i.e., an “open-loop” exercise test). This differs from situations in which ACT ingestion has been reported to be ergogenic, such as completion of a fixed-distance (16.1-km) time trial (30), a fixed number of maximal-effort repetitions (19, 31), or a fixed duration of maximal effort (32), all of which have a predetermined end point (i.e., a “closed-loop” exercise task). Moreover, since exercise-induced pain sensation is positively associated with exercise intensity (5, 13) and since ACT ingestion is suggested to be ergogenic by mitigating pain sensation (19, 30), this might account for the lack of improvement in performance in the longer-duration, continuous severe-intensity exercise test we employed compared with the improved exercise performance that has been reported during maximal-intensity exercise (19, 31, 32). With regard to contralateral fatigue development, we cannot exclude the possibility that ACT might have been effective at attenuating the effects of prior single-leg fatigue on T_lim_ during subsequent exercise if a greater degree of contralateral fatigue had been attained. For example, T_lim_ was lowered by 19% in Leg_2PL-CONTRA_ compared with Leg_2CON_ in the current study, whereas Amann et al. (3) reported a much larger (49%) reduction in T_lim_ following contralateral limb fatigue, which would have provided greater scope for an ergogenic effect with ACT ingestion. Moreover, since RPE is higher and T_lim_ is shorter at the same relative exercise intensity when a larger muscle mass is recruited (36), it is possible that ACT ingestion might have improved T_lim_ during exercise after prior fatigue, had a larger muscle mass been recruited in either the initial or the subsequent fatiguing exercise task. Further research is required to assess the exercise settings in which ACT administration is more or less likely to be ergogenic, including those with small compared with large muscle group exercise, in different exercise intensity domains, and with different pacing profiles (CWR compared with maximal and self-paced).

### Perspectives and Significance

The completion of prior single-leg fatiguing exercise compromised exercise tolerance during subsequent exercise in the contralateral leg. This ergolytic effect of prior contralateral leg fatigue was accompanied by elevated baseline RPE, muscle activation, and [ADP] and lower baseline [PCr], leading to the earlier attainment of peak (RPE, muscle activation, and [ADP]) or nadir (muscle [PCr]) values in these variables and attainment of a submaximal end-exercise [Pi]. However, acute ACT ingestion was not effective at lowering perceived exertion, increasing muscle activation or intramuscular perturbation, or enhancing T_lim_ during single-leg severe-intensity exercise completed with or without prior fatigue in the contralateral leg. These findings do not support an ergogenic effect of analgesia during severe-intensity single-leg dynamic contractions.

## GRANTS

This research was not sponsored by any funding body external to the University of Exeter. J. Fulford’s salary was supported via National Institute for Health Research Grant CRF/2016/10027 to the University of Exeter.

## DISCLOSURES

No conflicts of interest, financial or otherwise, are declared by the authors.

## AUTHOR CONTRIBUTIONS

P.T.M., S.J.B., A.V., and A.M.J. conceived and designed research; P.T.M., R.A.B., and J.F. performed experiments; P.T.M., R.A.B., and J.F. analyzed data; P.T.M., S.J.B., J.F., A.V., and A.M.J. interpreted results of experiments; P.T.M. and R.A.B. prepared figures; P.T.M., S.J.B., and A.M.J. drafted manuscript; P.T.M., S.J.B., J.F., and A.M.J. edited and revised manuscript; P.T.M., S.J.B., R.A.B., J.F., A.V., and A.M.J. approved final version of manuscript.
